# Albinism: Images in ophthalmology

**DOI:** 10.4103/0974-620X.48423

**Published:** 2009

**Authors:** O. K. Sreelatha, E. Al-Harthy, P. VanRijen-Cooymans, S. Al-Zuhaibi, A. Ganesh

**Affiliations:** Department of Ophthalmology, Sultan Qaboos University Hospital, Muscat, Oman

## Introduction

An 8-year-old child, daughter of Omani parents, presented to the pediatric ophthalmology clinic of Sultan Qaboos University Hospital, Oman, with defective vision, photophobia, and rapid movements of both eyes since birth. The child was otherwise healthy. Both parents were healthy and the marriage was first degree consanguineous. There was a positive family history of similar problems in the patient’s younger brother and paternal aunt. Clinical examination concluded with the diagnosis of oculocutaneous albinism. The patient’s sibling was also examined and diagnosed with the same condition.

## Comment

Albinism (origin - Latin word albus which means white) refers to a group of hereditary disorders with an abnormality of melanin synthesis or distribution. The incidence of oculocutaneous albinism in Oman is 1 in 30,000 live births.[1] There are various types of albinism [Table 1]. The main clinical features are hypopigmentation of hair, skin, and eyes. Ophthalmic manifestations include photophobia, nystagmus, defective vision, and squint. The visual acuity in patients with albinism usually is poor and ranges from 20/60 to 20/400. Visual prognosis is usually guarded. However, the children with albinism grow and develop normally and reach normal intelligence levels.[2] The visual-evoked potential test (VEP) is a sensitive diagnostic modality for confirming the diagnosis of albinism. Patients with albinism show an asymmetry of VEP between the two eyes secondary to misrouting of optic pathways.[3] Optical coherence tomography (OCT) is useful in identifying foveal hypoplasia in patients with albinism.[4]

**Table 1:** Common types of albinism with brief description of characteristics

Types of albinism	Subdivision	Subdivision	Etiological factor	Manifestations	Signs and symptoms	Inheritance/trait
Oculo-cutaneous	OCA1*	OCA1A	Tyrosinase negative	Absence of skin	1. Skin, hair and eye discoloration [Figure 1]	Autosomal recessive disorder
				or ocular pigment	2. Tendency to sunburn easily	(affects both sexes equally)
					3. Photophobia due to pigmentary	
					abnormalities of iris and retina	
					4. defective vision	
					5. Pendular nystagmus	
					6. Strabismus, mostly esotropia	
					7. Refractive error	
					8. Red pupil [Figure 2)	
					9. Iris trans- illumination [Figure 3]	
					10. Foveal hypoplasia [Figure 4]	
						
					11. Optic nerve hypoplasia	
					12. Abnormal decussation of the optic	
					nerve fi bers	
		OCA1B	Tyrosinase positive	Can develop some		
				pigment during		
				the growth		
	OCA2*		Defect in the P protein	Minimal amount		
				of pigment		
	OCA3*		Defect in TYRP1 protein	Substantial pigment		
	OCA4*	Defect in SLC45A2	Minimal amount			
			protein	of pigment		
Syndromic albinism	Hermansky–	Other genes	Hypo pigmentation,	Signs and symptoms 1 to 12		
		Pudlak	involvement	bleeding problems and,	as above and systemic features	
		syndrome		cellular storage disorders		
		Chediak –	Other genes	Hypo pigmentation and		
		Higashi	involvement	defect in white blood cells,		
		syndrome		prone to infections		
Ocular albinism	OA 1*		Defect of the	Absence of ocular pigment	Only ocular symptoms and	X-linked recessive (affects
			GPR143 gene		signs (3 to 12 as above) and	males) Autosomal recessive
					mosaic fundal pigmentation	(affects both sexes equally)
	OA 2*		Not exactly known	Absence of ocular	Only ocular symptoms and	
				pigment	signs (3 to 12 as above)	
					and color blindness	
	AROA*		Defect in	Absence of	Only ocular symptoms and	
			chromosome 6	ocular pigment	

* OCA - Oculocutaneous albinism

* OA - Ocular albinism

* AROA - Autosomal Recessive Ocular Albinism,

There is no medical cure for albinism. Supportive care includes: 1] Periodic examination of patients to monitor their visual development and to assess the status of their refractive error and/or strabismus. 2] Vision rehabilitation by correction of refractive errors, use of filter/tinted glasses and caps/visors and prescription of low visual aids and vision enhancing tips. 3] Skin care: There is an increased risk of skin cancer in these patients. They should be advised to use skin-tanning lotion with Sun Protection Factor of 15 or greater and proper clothing for protection against exposure to sunlight. 4] Referral to appropriate sub-specialists for systemic evaluation. 5] Genetic testing and counseling for patients and their families. 6] Counseling by a social worker for social support and to dispel existing myths about albinism.

**Figure 1:**
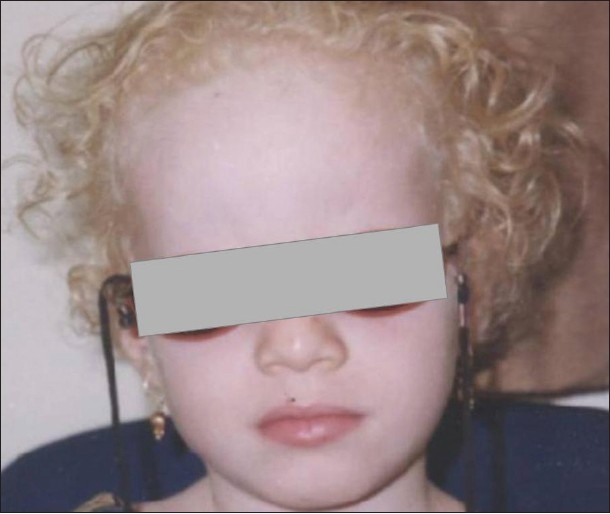
Face photo of an Omani girl shows external features of oculocutaneous albinism. Note blond hair and white skin

**Figure 2:**
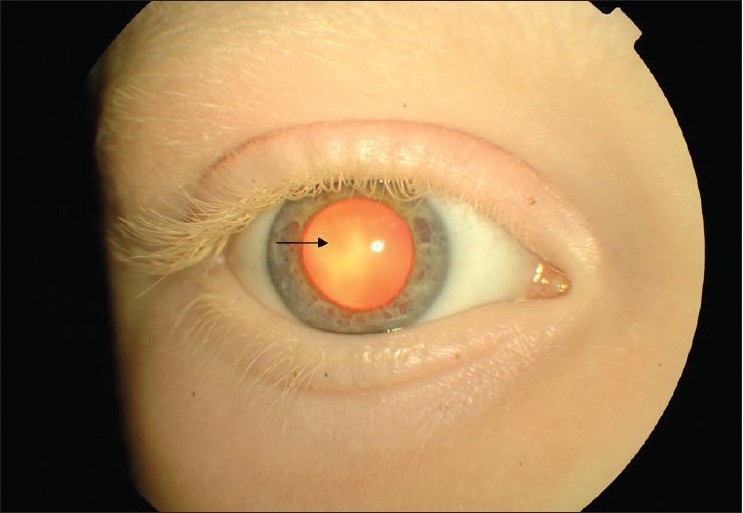
Red pupil (arrow) due to the poor absorption of light from hypo-pigmented retina. Also note hypo-pigmented eye lashes, eye brows, and light iris

**Figure 3:**
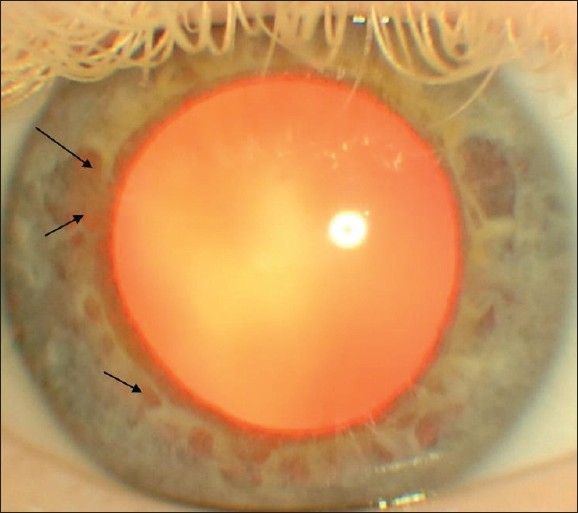
Iris transillumination (arrows) due to escape of refl ected light from the retina through the iris

**Figure 4:**
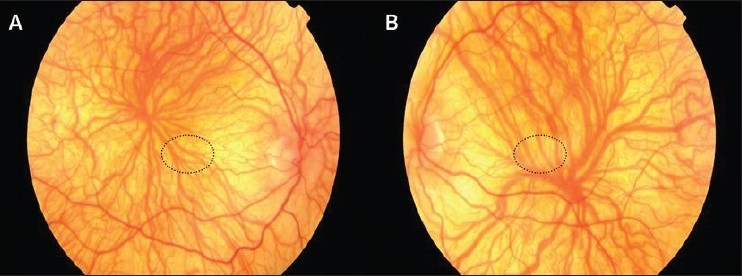
Fundus photo of right eye (A) and left eye (B); shows clear view of choroidal vasculature due to the hypo-pigmentation of retinal pigment epithelium, pale retina, foveal hypoplasia, and indistinct optic disc margin. Dotted circle shows the location of normal fovea.
